# Resistance to Clarithromycin and Fluoroquinolones in *Helicobacter pylori* Isolates: A Prospective Molecular Analysis in Western Romania

**DOI:** 10.3390/antibiotics14121223

**Published:** 2025-12-04

**Authors:** Patricia Serena, Ruxandra Mare, Bogdan Miutescu, Renata Bende, Alexandru Popa, Giovanni Aragona, Edward Seclăman, Luca Serena, Andreea Barbulescu, Roxana Sirli

**Affiliations:** 1 Division of Gastroenterology and Hepatology, Department of Internal Medicine II, “Victor Babes” University of Medicine and Pharmacy Timisoara, Eftimie Murgu Square 2, 300041 Timisoara, Romania; patricia.lupulescu@umft.ro (P.S.); miutescu.bogdan@umft.ro (B.M.); bende.renata@umft.ro (R.B.); popa.alexandru@umft.ro (A.P.); sirli.roxana@umft.ro (R.S.); 2 Advanced Regional Research Center in Gastroenterology and Hepatology, “Victor Babes” University of Medicine and Pharmacy Timisoara, 300041 Timisoara, Romania; 3 Gastroenterology and Hepatology Unit, Guglielmo da Saliceto Hospital, 29121 Piacenza, Italy; g.aragona@ausl.pc.it; 4 Department IV—Biochemistry and Pharmacology, Faculty of Medicine, “Victor Babes” University of Medicine and Pharmacy Timisoara, Eftimie Murgu Square 2, 300041 Timisoara, Romania; eseclaman@umft.ro; 5 Anesthesia and Intensive Care Department, Guglielmo da Saliceto Hospital, 29121 Piacenza, Italy; l.serena@ausl.pc.it; 6 Gastroenterology and Hepatology Unit, CF Clinical Hospital, 300173 Timisoara, Romania; barbulescu.andra91@gmail.com

**Keywords:** *Helicobacter pylori*, antibiotic resistance, molecular testing

## Abstract

**Background and Objectives:** *Helicobacter pylori* (*H. pylori*) infection remains one of the most common chronic bacterial infections worldwide and is associated with a wide range of gastrointestinal disorders, including gastritis, peptic ulcer disease, and gastric cancer. Increasing rates of antibiotic resistance, particularly to clarithromycin and fluoroquinolones, represent a major therapeutic challenge. The objective of this study was to determine the prevalence of resistance-associated mutations in *H. pylori*-positive gastric biopsy samples from western Romania. **Materials and Methods:** We conducted a prospective study from January to December 2024, enrolling 138 patients undergoing gastroscopy. Biopsies were collected from the gastric antrum, and *H. pylori* infection was confirmed using the rapid urease test (RUT). Positive samples were further analyzed with the GenoType HelicoDR assay to detect mutations in the *23S rRNA* gene (clarithromycin resistance) and *gyrA* gene (fluoroquinolone resistance). Clinical, demographic, and endoscopic data were also collected. **Results:** *H. pylori* infection was confirmed in 41.3% of the patients (57), of whom 63.2% (36) were treatment-naïve and 36.8% (21) had prior eradication therapy. Among treatment-naïve patients, clarithromycin resistance was identified in 19.4%, whereas previously treated patients showed a markedly higher resistance rate of 47.6% (*p* = 0.018). All clarithromycin-resistant cases carried the A2147G (*23S* MUT3) mutation. Fluoroquinolone resistance was present in 13.9% of naïve patients and increased to 23.8% in those with prior eradication therapy, with resistance linked to *gyrA* mutations at codons 87 (N87K) and 91 (D91 variants). Combined resistance to both antibiotics was observed only in a subset of previously treated patients. **Conclusions:** Primary resistance to clarithromycin in western Romania exceeds the 15% threshold defined by international guidelines, making clarithromycin-based triple therapy unsuitable as an empirical first-line option. The findings support the use of bismuth quadruple therapy as the preferred empirical regimen in this region. Also, molecular testing proved effective for rapid identification of resistance-associated mutations.

## 1. Introduction

*Helicobacter pylori* (*H. pylori*) is a Gram-negative, spiral-shaped bacterium that colonizes the gastric mucosa and is recognized as a class I carcinogen by the World Health Organization [1]. This microorganism is responsible for a wide range of gastrointestinal disorders, including chronic gastritis, peptic ulcer disease, and gastric cancer [2].

The prevalence of *H. pylori* varies significantly worldwide, with infection rates influenced by socioeconomic conditions, hygiene practices, and healthcare access [3]. A systematic review and meta-analysis estimated the global prevalence at 44.3%, with higher rates reported in developing countries compared with industrialized nations. For instance, infection rates in Latin America are as high as 59.3%, whereas in North America, they are as low as 25.8% [3]. Recent European data report heterogeneous prevalence patterns, with estimates ranging from 16.8% in Northern Italy to 32% in Southeastern Hungary and 35.8% in Poland [4,5,6]. In Romania, prevalence studies suggest infection rates of approximately 40%, underscoring the regional burden of this pathogen [7,8].

The optimal strategy for managing *H. pylori* infection remains a subject of debate. An effective anti-*H. pylori* treatment is currently defined as one that consistently achieves a cure rate of at least 90%, which has become an accepted benchmark. Due to a significant decline in effectiveness, with eradication rates dropping below 80–85%, triple therapy comprising a proton pump inhibitor (PPI), clarithromycin (CLA), and either amoxicillin or metronidazole is generally no longer recommended as an empirical first-line treatment for *H. pylori* infection, except in regions where CLA resistance is known to be below 15%. This change in recommendations is mainly driven by the worldwide increase in CLA resistance, which directly compromises the efficacy of triple therapy [9,10].

As a result, recent guidelines, including the Maastricht VI/Florence consensus, advocate for bismuth-based quadruple therapy as the preferred first-line treatment in such settings. This regimen includes a PPI, bismuth, tetracycline, and metronidazole, and consistently achieves eradication rates exceeding 90% in most studies [11].

Despite the availability of effective therapies, the increasing prevalence of antibiotic-resistant *H. pylori* poses a significant challenge to eradication efforts [12]. Routine susceptibility testing prior to prescribing first-line *H. pylori* treatment can help assess antibiotic resistance and improve treatment effectiveness [13]. Molecular testing for *H. pylori* plays an essential role in managing antibiotic resistance and guiding tailored treatments. Techniques such as polymerase chain reaction (PCR)-based assays and next-generation sequencing (NGS) have gained prominence in recent years as alternatives to traditional culture-based testing [9]. These advanced methods are widely used to detect genetic mutations associated with resistance. Compared with traditional microbiological approaches such as culture and phenotypic susceptibility testing, molecular methods offer several key advantages. Culture of *H. pylori* is technically demanding, time-consuming, and often unsuccessful due to the fastidious nature of the organism, whereas PCR-based assays achieve high sensitivity and provide rapid results [14,15]. Moreover, PCR-based assays allow identification of the key mutations responsible for CLA and fluoroquinolone (FQ) resistance with high accuracy, while NGS offers a broader genomic assessment, detecting additional or rare mutations and even mixed susceptible–resistant populations that culture frequently misses [16,17,18]. The ability to perform these tests directly on gastric biopsy or stool samples has facilitated the implementation of susceptibility-guided therapies, improving eradication success rates [19,20]. For instance, the GenoType HelicoDR assay identifies resistance-associated mutations with sensitivity exceeding 90% when applied to biopsy samples [14,21].

CLA resistance, a primary contributor to treatment failure, is largely caused by point mutations in the *23S rRNA* gene, specifically A2143G, A2142G, and A2142C [22]. These mutations alter the binding site of CLA, decreasing its efficacy. The mutations are commonly identified using PCR-based assays or NGS, both of which have shown high sensitivity and specificity [23]. FQ resistance is primarily due to point mutations in the *gyrA* gene at codons 87 and 91, which encode DNA gyrase subunits critical for bacterial DNA replication [24]. These mutations lead to decreased drug binding and reduced efficacy. Resistance rates for FQ, particularly levofloxacin, are rising globally, with rates surpassing 20% in several regions, necessitating caution in their use as second-line therapies [1,25]. These resistance-associated mutations have important public health consequences. By reducing antibiotic binding and compromising treatment efficacy, *23S r*RNA and *gyrA* mutations allow the infection to persist, maintaining chronic gastric inflammation and contributing to the development of complications. Resistant *H. pylori* isolates are more difficult to eradicate, which increases the likelihood of recurrent treatment attempts and facilitates continued transmission within the community. At a population level, these trends contribute to higher healthcare costs and a growing disease burden [26,27].

The aim of this study was to evaluate the prevalence of CLA and FQ resistance in *H. pylori*-positive gastric biopsy samples. Utilizing molecular diagnostic methods, the study focused on detecting resistance-associated mutations in the *23S rRNA* and *gyrA* genes. By identifying these mutations and assessing local resistance patterns, the study aims to provide critical insights for tailoring evidence-based eradication therapies and improving treatment outcomes for patients with *H. pylori* infections.

## 2. Results

### 2.1. Baseline Clinical and Endoscopic Characteristics of the Study Population

**Figure 1:**
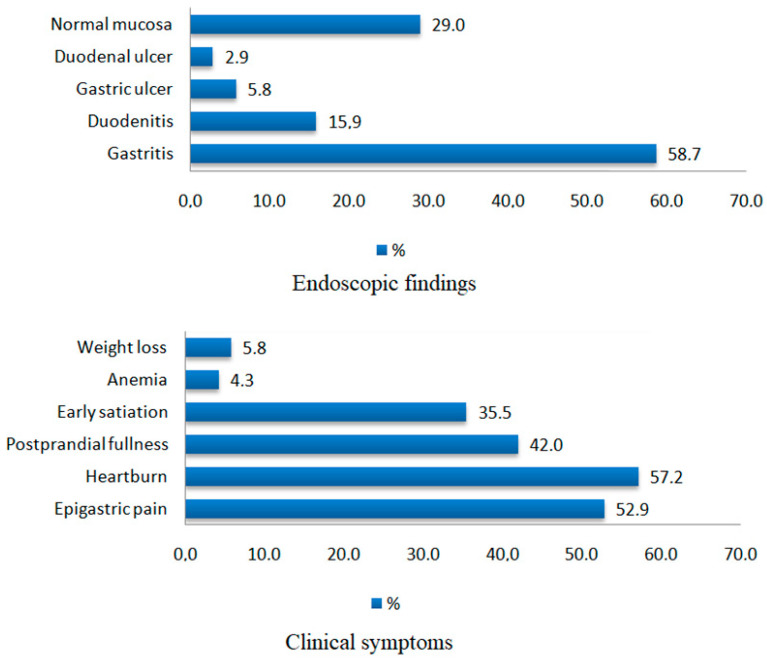
Endoscopic findings and clinical symptoms in the study population (*n* = 138).

The study included a cohort of 138 patients, with a mean age of 50.2 ± 13.4 years and a median of 51 years (20–79), of whom 54.3% (75) were female. The treatment-naïve group consisted of 104 patients (54 women, 51.9%; 50 men, 48.1%), with a mean age of 50.27 ± 13.57 years and a median of 52 years (20–79), while the previously treated group included 34 patients (21 women, 61.8%; 13 men, 38.2%), with a mean age of 51.76 ± 12.83 years and a median of 52 years (24–71). Regarding clinical presentation, the distribution of gastrointestinal symptoms was broadly similar between the two groups, with no statistically significant differences observed. Epigastric pain was more frequently reported in treatment-naïve patients (56.7%) compared with previously treated patients (41.2%), although this difference did not reach statistical significance (*p* = 0.168). Heartburn was present in 59.6% of naïve patients and 50.0% of experienced patients (*p* = 0.433). Postprandial fullness showed a similar distribution between groups, occurring in 41.3% of naïve patients and 44.1% of previously treated patients (*p* = 0.933). Early satiation was reported in 32.7% of naïve patients and 44.1% of experienced patients, with no significant difference (*p* = 0.316). Anemia was uncommon in both groups, occurring in 4.8% of naïve patients versus 2.9% of previously treated patients (*p* = 1.000). Weight loss was reported by 6.7% of naïve patients and 2.9% of experienced patients, also without a statistically significant difference (*p* = 0.690). The distribution of the main clinical symptoms and endoscopic findings among the study population is illustrated in Figure 1. 

*H. pylori* infection was confirmed in 41.3% (57) patients using the rapid urease test (RUT). All RUT-positive biopsies were subsequently analyzed by molecular testing to assess CLA and FQ resistance. Of the 57 positive cases, 63.2% (36) patients had no prior history of eradication therapy and were classified as naïve, while the remaining 36.8% (21) patients had previously undergone treatment for *H. pylori* infection.

### 2.2. Resistance to CLA (23S rRNA Mutations)

In the overall *H. pylori*-positive cohort (*n* = 57), CLA resistance was found in 29.8% (17) of the patients whereas 70.2% (40) presented CLA-susceptible profiles. Among naïve patients (*n* = 36), 80.6% (29) showed CLA sensitive genotypes and 19.4% (7) exhibited CLA-resistant genotypes. In previously treated patients (*n* = 21), 52.4% (11) had CLA-susceptible profiles and 47.6% (10) displayed CLA-resistant profiles. The difference in resistance rates between naïve and previously treated groups was statistically significant (*p* = 0.018).

**Figure 2:**
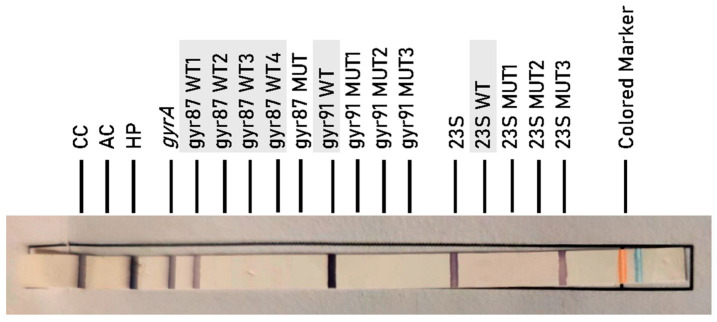
CLA-resistant *H. pylori* specimen with *23S* MUT3 profile determined by GenoType HelicoDR.

In both treatment-naïve and previously treated groups, all CLA-resistant specimens exhibited the *23S* MUT3 genotype, corresponding to the A2147G substitution, as exemplified in Figure 2. No specimen carried MUT1 or MUT2 mutations.

The genotype profiles identified in both groups are summarized in Table 1.

### 2.3. Resistance to FQ (gyrA Mutations)

In the *H. pylori*-positive cohort (*n* = 57), FQ resistance was detected in 13.9% (5/36) of treatment-naïve patients and in 23.8% (5/21) of previously treated patients, yielding an overall prevalence of 17.5% (10/57). FQ-sensitive cases were significantly more frequent than resistant ones in the naïve group (86.1% vs. 13.9%, *p* < 0.0001) and also in the previously treated group (76.2% vs. 23.8%, *p* = 0.0020). 

Among the naïve patients (*n* = 36), the five FQ-resistant specimens exhibited three distinct *gyrA* mutation patterns. Three specimens carried the *gyr*87MUT profile, corresponding to the N87K mutation. One specimen harbored the *gyr*91MUT1 profile, associated with the D91N substitution. Another specimen showed a heterogeneous genotype at codon 87, with hybridization signals for both *gyr*87WT2 and *gyr*87MUT (N87K), suggesting either heteroresistance or the presence of a mixed bacterial population. 

Similarly, in the previously treated group (*n* = 21), five FQ-resistant specimens were identified. Two of them exhibited the *gyr*87MUT profile, corresponding to the N87K mutation in the *gyrA* gene and one specimen carried the *gyr*91MUT1 profile, associated with the D91N substitution. As observed in the naïve group, heterogeneity at codon 87 was also present in this group, with two specimens showing mixed wild-type and mutant hybridization signals (*gyr*87WT1 and *gyr*87MUT) suggestive of heteroresistance.

**Figure 3:**
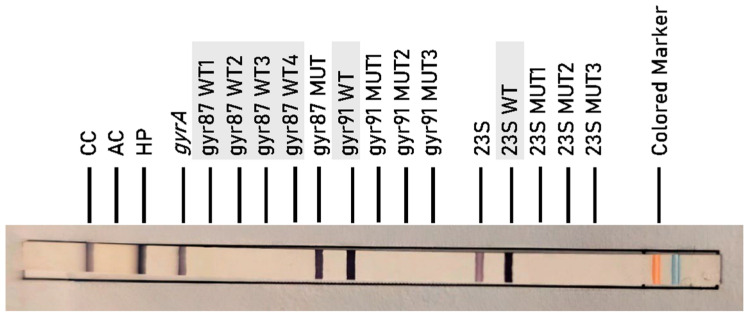
FQ-resistant *H. pylori* specimen with *gyr*87MUT profile determined by GenoType HelicoDR.

An example of FQ-resistant *H. pylori* isolate carrying the *gyr*87MUT profile, as determined by the GenoType HelicoDR assay, is illustrated in Figure 3 and the genotype profiles identified in both groups (naïve and previously treated) are summarized in Table 2.

**Figure 4:**
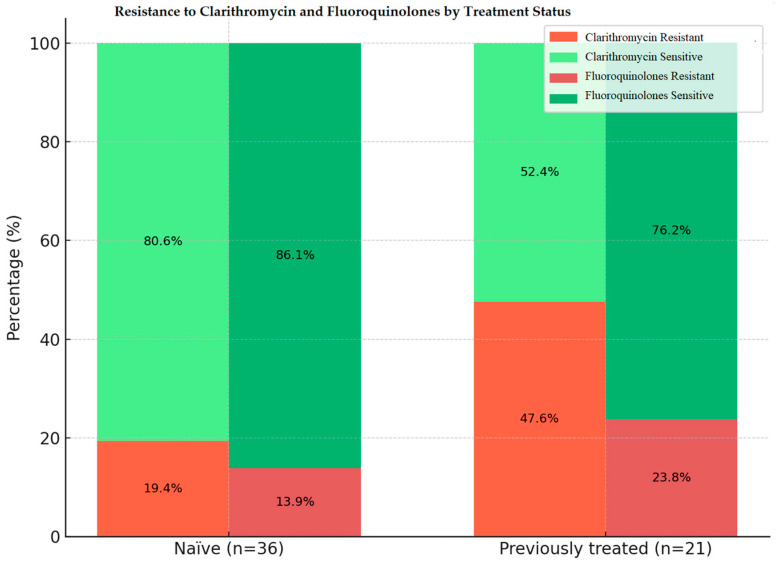
Comparative resistance to CLA and FQ in treatment-naïve versus previously treated *H. pylori*-positive patients.

The distribution of CLA and FQ resistance rates, stratified by treatment status (treatment-naïve vs. previously treated patients), together with within- and between-group statistical comparisons, are summarized in Table 3, while a comparative visual representation of sensitivity (green) and resistance (red) in the two groups is provided in Figure 4.

### 2.4. CLA and FQ Resistance Patterns by Treatment Status

In the group of previously treated patients (*n* = 21), dual resistance to both CLA and FQ was identified in 9.5% (2/21) of the cases. The *H. pylori* isolates from these two patients showed distinct resistance genotypes: one isolate carried the *gyr*87MUT profile (codon 87 mutation N87K), while the other exhibited the *gyr*91MUT1 profile (D91N substitution), both associated with FQ resistance. In addition, both isolates carried the *23S* MUT3 (A2147G) substitution responsible for CLA resistance. 

In the treatment-naïve group (*n* = 36), no cases of dual resistance were detected.

## 3. Discussion

### 3.1. Current Findings and Literature Findings

In our study, the prevalence of *H. pylori* infection among symptomatic patients was 41.3%, as confirmed by RUT. This rate reflects the current local burden of infection in Western Romania and aligns with previously reported values from other Romanian regions. Recent epidemiological studies have reported regional variations in *H. pylori* prevalence, ranging from approximately 40% in Northwestern Romania [28] to over 60% in the southern region [29]. These regional differences are likely multifactorial and reflect underlying disparities in socioeconomic status, hygiene conditions, and access to healthcare, factors consistently associated with higher *H. pylori* transmission in several epidemiological studies [30].

Regarding antibiotic resistance, we found that primary resistance to CLA was 19.4%, whereas resistance among previously treated patients increased to 47.6% (*p* = 0.018). For FQ, primary resistance was 13.9%, and higher (23.8%) in the previously treated group (*p* = 0.557). Importantly, heteroresistance (simultaneous detection of susceptible and resistant isolates in gastric biopsies from a single patient) was detected for *gyrA* mutations, including N87K, and in some cases was associated with concurrent CLA resistance (A2147G). Our results are consistent with large European surveillance projects. The European Registry on *H. pylori* Management (Hp-EuReg) reported primary resistance rates of approximately 25% for CLA and around 20% for levofloxacin, with higher prevalence in Southern and Eastern Europe compared to Northern countries [31]. Priadko et al. conducted a prospective study in France in 2025, evaluating primary CLA resistance by both culture and RT-PCR; resistance was identified in 17.7% and 18.7% of isolates, respectively [32]. A multicenter Italian study conducted by Saracino et al. between 2009 and 2019 evaluated antibiotic resistance in treatment-naïve patients and demonstrated a significant increase in primary CLA resistance from 30.2% in 2009–2014 to 37.8% in 2015–2019, and in primary levofloxacin resistance from 25.6% to 33.8% over the same period [33]. In contrast, surveillance programs in Northern European countries continue to show much lower levels, with primary CLA and levofloxacin resistance frequently below 10% [34]. In Romania, recent molecular and culture-based investigations have provided complementary data on *H. pylori* resistance patterns. A multicenter study conducted in Northwestern and Central regions in 2023 reported primary resistance rates of 16.7% for CLA and 11.1% for FQ, while secondary resistance reached 75.8% and 30.3%, respectively [35].

At the global level, marked heterogeneity in *H. pylori* resistance has been documented. A systematic review and meta-analysis by Savoldi et al., including 178 studies and over 66,000 isolates from 65 countries, reported that primary CLA resistance exceeded 15% in most WHO regions, reaching 18% in Europe, 33% in the Eastern Mediterranean, and 34% in the Western Pacific. By contrast, it remained lower in the Americas (10%) and Southeast Asia (10%). Primary levofloxacin resistance was reported at 11% in Europe but ≥20% in several Asian regions. The same analysis demonstrated that secondary resistance was consistently higher, with CLA resistance rising to 48% in Europe and up to 67% in the Western Pacific [27]. Evidence from Southeast Asia further highlights this issue: Dang et al. conducted a prospective study in Vietnam and found primary CLA resistance of 66.1% and secondary resistance of 94.3%, while primary and secondary levofloxacin resistance reached 38.1% and 48.6%, respectively [36]. These variations are strongly associated with regional antibiotic use, particularly widespread prescriptions of macrolides for respiratory infections and FQ for urinary tract infections [34,37]. Overall, the marked discrepancies in resistance patterns observed across continents and even within individual regions emphasize the importance of documenting local prevalence rates, since knowledge of regional resistance remains essential for interpreting guideline recommendations and for optimizing empirical treatment choices [38]. Our findings have direct therapeutic implications. Since our study demonstrated that primary CLA resistance exceeded the 15% threshold, CLA-based triple therapy cannot be recommended as an empirical first-line regimen. According to the Maastricht VI/Florence Consensus Report (2022), the recommended first-line empirical treatment in areas of high CLA resistance (>15%) is a 14-day bismuth quadruple therapy [11].

Molecular methods offer clear advantages for resistance detection in *H. pylori*. In our study, the GenoType HelicoDR assay was applied to RUT-positive biopsies to assess antibiotic resistance. We used this test specifically for determining resistance patterns rather than for primary diagnosis because RUT served as the initial detection method in our clinical workflow. Restricting molecular testing to positive samples allowed us to use resources efficiently and ensure a cost-effective approach, while still meeting the study objective. The GenoType HelicoDR assay has been extensively validated as a molecular tool for the simultaneous detection of CLA and FQ-associated mutations in *H. pylori* [21]. The assay has been previously evaluated in comparison with histology and culture and demonstrated high sensitivity and specificity for *H. pylori* detection, while also accurately identifying resistance to CLA and FQ [39]. Subsequent studies confirmed its reliability and clinical utility, demonstrating high agreement with phenotypic data and highlighting its ability to detect heteroresistant specimens that may be overlooked by culture [40]. In comparison with other molecular approaches, the HelicoDR assay offers several practical advantages: it provides simultaneous detection of *H. pylori* and the key resistance mutations within a single workflow, has a short turnaround time, and does not require advanced sequencing platforms. These features make it suitable for routine use in laboratories that may not have access to real-time PCR or NGS facilities [41]. However, as described by Cambau et al., the HelicoDR assay has a few inherent technical limitations. Because the strip includes probes specifically targeting the most prevalent mutations in the *23S rRNA* and *gyrA* genes, some substitutions may fall outside the targeted regions. In their evaluation, isolates showing no WT/MUT band at codon 87 were found to carry a sequence variation near codon 88 of the *gyrA*, a change that did not confer FQ resistance but weakened probe hybridization and produced atypical banding patterns [21]. Recent genomic studies have also identified additional *gyrA* mutations associated with levofloxacin resistance, including A88V/P and N97K, which are not covered by the HelicoDR probe set. Taken together, these findings show that the HelicoDR assay is a reliable and practical tool for rapid detection of the most common resistance-associated mutations [15].

At the genotypic level, all CLA-resistant isolates in our series carried the A2147G substitution in the *23S r*RNA (MUT3). The most prevalent and well-characterized mutations in the *23S r*RNA gene are adenine-to-guanine transitions at positions 2146 (A2146G) and 2147 (A2147G), or, less frequently, an adenine-to-cytosine transversion at position 2146 (A2146C). Together, these substitutions account for more than 90% of CLA resistance in *H. pylori* isolates from developed countries [22]. This observation is consistent with reports from other molecular studies, which identified A2147G (equivalent to A2143G in alternative numbering) as the most common CLA-resistance mutation, whereas A2146G/C are less frequently encountered [42,43]. A recent European comparative study of molecular methods reported that among 26 CLA-resistant isolates, 61.5% carried the A2147G mutation, 11.5% had A2146G, and heteroresistant profiles included both A2146C and A2147G in combination with wild type sequences [44].

Mutations at *gyrA* codons 87 and 91 (such as N87K and D91G/N/Y) remain the most frequent molecular signatures associated with FQ resistance in *H. pylori*, as confirmed by recent studies [45]. In our cohort, FQ resistance was linked exclusively to *gyr*91MUT1 profile (associated with the D91N substitution) and to *gyr*87MUT profile (corresponding to the N87K mutation). Neither the *gyr*91MUT2 (D91G) nor the *gyr*91MUT3 (D91Y) variants were detected in any of the analyzed isolates. This pattern is consistent with large European surveillance data. Garcia et al. conducted a study in France analyzing 97 FQ-resistant *H. pylori* isolates and reported D91N as the most prevalent codon 91 mutation, whereas D91G and D91Y occurred far less frequently, supporting the distribution observed in our series [46].

Two cases in our research showed resistance to both CLA and FQ: one specimen carried the *gyr*87MUT (N87K) profile together with the *23S* MUT3 substitution, while the other exhibited the *gyr*91MUT1 (D91N) profile combined with *23S* MUT3. This combination is consistent with previously reported multidrug-resistant *H. pylori* genotypes. Although these mutations do not directly increase virulence, they facilitate bacterial persistence by reducing antibiotic binding, thereby prolonging gastric inflammation and contributing to the development of complications. The circulation of such resistant isolates increases the likelihood of treatment failure, the need for repeated eradication attempts, and the overall healthcare burden [26,27].

Heteroresistance, reflected by the simultaneous detection of susceptible and resistant specimens in gastric biopsies from a single patient, is not uncommon. In recent years, this phenomenon has posed significant challenges to *H. pylori* eradication, yet its characteristics remain insufficiently understood. In clinical practice, heteroresistance allows resistant subpopulations to persist despite standard therapies, increasing the risk of ongoing infection and the need for repeated eradication attempts [47]. With the increasing prevalence of heteroresistance and the concomitant decline in eradication rates, it is essential to optimize detection methods and elucidate the underlying resistance mechanisms in order to support the development of more precise and effective treatment strategies [48]. Recently Wang et al. reported *gyrA* heteroresistance in 14.3% of gastric biopsy specimens, most frequently involving combinations of wild-type and mutant alleles at positions 87 and 91 [49]. We also observed heteroresistant genotypes in our cohort. Specifically, three isolates exhibited mixed hybridization signals for the N87K mutation, showing both wild-type (*gyr*87WT1 or *gyr*87WT2) and mutant (*gyr*87MUT) bands. For CLA, however, no mixed WT/MUT profiles were detected in our cohort, although it has been documented in the literature. Kocsmár et al. conducted a study in Hungary involving 305 patients with *H. pylori* infection and reported CLA heteroresistance in 12.5% of cases [50]. The absence of similar patterns in our cohort is most likely related to cohort size. Future studies should aim to characterize the prevalence and clinical impact of heteroresistance in larger populations and evaluate whether incorporating targeted susceptibility testing in selected cases improves eradication rates.

### 3.2. Study Limitations

This single-center prospective study offers valuable insight into local *H. pylori* resistance patterns in western Romania. A key limitation of the study is that resistance testing was performed exclusively using molecular methods, without confirmation by bacterial culture. Future research should involve larger patient cohorts, and longitudinal follow-up studies should be conducted to assess the relationship between identified mutations and treatment outcomes.

## 4. Materials and Methods

### 4.1. Research Design and Ethical Consideration

A prospective study was conducted from January to December 2024, enrolling 138 patients undergoing gastroscopy at a tertiary healthcare facility in western Romania. Data collection included demographic characteristics, clinical presentations, endoscopic findings, rapid urease test (RUT) status and molecular results identifying *H. pylori* resistance-associated mutations. The research followed the ethical principles outlined in the 1975 Declaration of Helsinki and received approval from the institution’s internal review board prior to initiation. Written informed consent was obtained from all participants.

### 4.2. Participant Selection and Sample Collection

The study included patients aged 18 years or older who underwent gastroscopy and RUT for the detection of *H. pylori*. The indications for gastroscopy covered a variety of upper gastrointestinal complaints, such as persistent or recurrent epigastric pain, heartburn, postprandial fullness, early satiation. Other indications included anemia caused by suspected gastrointestinal bleeding and unexplained weight loss.

Exclusion criteria included patients younger than 18 years, those with decompensated hepatic, cardiac, pulmonary, or renal diseases, severe mental health disorders, pregnancy, breastfeeding, known malignancy, and gastric cancer. These criteria ensured a clinically relevant and homogenous study cohort.

All endoscopic procedures were performed under anesthesiological supervision. Sedation was achieved using midazolam, fentanyl, and propofol, according to the internal anesthesia protocols of our institution. Gastroscopic examinations were performed using Olympus Evis EXERA III (CV-190) and GIF-HQ190 endoscopes (Olympus Corp., Tokyo, Japan). Biopsies were collected from the gastric antrum and corpus and RUT was performed using the AMA RUT Pro test (AMA-Med Oy, Mikkeli, Finland). The test contains a reactive medium composed of urea and a pH indicator. Biopsy samples were placed directly into this medium and monitored for the characteristic color shift from yellow to red, indicating urease activity consistent with *H. pylori* infection. Results were interpreted at the recommended 5 min reading time. The AMA RUT Pro test has high sensitivity (99.25%) and specificity (98.76%). Biopsy samples that tested positive on RUT were then placed in sterile microtubes without fixatives, transported promptly to the laboratory, kept refrigerated (2–8 °C), and processed within 24–48 h for molecular analysis to identify resistance-associated mutations to CLA and FQ. Additionally, all endoscopic findings were documented, including gastritis, duodenitis, gastric ulcer, duodenal ulcer and other pathological observations.

### 4.3. Molecular Testing

Biopsy specimens collected were processed for DNA extraction using a validated tissue protocol QIAamp DNA Mini Kit (Qiagen, Hilden, Germany) according to manufacturer’s protocol.

Molecular detection of *H. pylori* resistance-associated mutations was performed using the GenoType HelicoDR assay (Hain Lifescience, Nehren, Germany). This DNA-STRIP-based assay allows for the identification of mutations in the *23S r*RNA gene (associated with CLA resistance) and the *gyrA* gene (associated with FQ resistance) directly from gastric biopsy specimens. These genomic regions are included in the assay because they contain the primary and well-established molecular mechanisms responsible for CLA and FQ resistance in *H. pylori*.

The *H. pylori* testing procedure followed the manufacturer’s protocol.

Banding patterns were interpreted using the kit’s standardized template. Each strip includes control zones for conjugate reaction (CC), amplification (AC), and locus-specific controls for both target genes (*gyrA* and *23S*). The presence or absence of wild-type (WT) and mutation-specific (MUT) bands determines susceptibility or resistance.

CLA Resistance (*23S rRNA* gene mutations):

- *23S* MUT1: corresponds to mutation A2146G.
- *23S* MUT2: corresponds to mutation A2146C.
- *23S* MUT3: corresponds to mutation A2147G.

FQ Resistance (*gyrA* gene mutations):

- *gyr*87 MUT: corresponds to mutations at codon 87-N87K.
- *gyr*91 MUT1: corresponds to mutation D91N.
- *gyr*91 MUT2: corresponds to mutation D91G.
- *gyr*91 MUT3: corresponds to mutation D91Y.

### 4.4. Statistical Analysis

All statistical analyses were performed using MedCalc Version 19.4 (MedCalc Software Ltd., Ostend, Belgium) and Microsoft Excel 2019 (Microsoft Corporation, Redmond, WA, USA). The dataset included demographic variables (age, sex), clinical characteristics (indication for gastroscopy, treatment status), and molecular results for H. pylori resistance markers (*23S* rRNA and *gyr*A mutations). Prior to analysis, data were checked for completeness, outliers, and coding accuracy.

Descriptive statistics were used to summarize the distribution of variables: continuous variables were assessed for normality using the Kolmogorov–Smirnov test and presented as mean ± standard deviation (SD) when normally distributed, or as median and interquartile range (IQR) when non-normally distributed. Categorical variables were reported as frequencies and percentages.

Comparisons between treatment-naïve and previously treated groups were performed using independent-samples Student’s *t*-tests for continuous variables and chi-square or Fisher’s exact tests for categorical variables where appropriate. Because some resistance patterns had very small frequencies (e.g., dual resistance), no multivariable modeling or regression analysis was performed, as such analyses would not yield reliable estimates.

A *p*-value < 0.05 was considered statistically significant for all analyses.

## 5. Conclusions

This study shows that CLA and FQ resistance remain major issues in western Romania. CLA resistance exceeded 15%, supporting bismuth-based quadruple therapy as the preferred first-line regimen. Molecular testing, such as the GenoType HelicoDR assay, enables rapid detection of resistance and can help guide personalized treatment, particularly in patients with previous eradication failure. These findings highlight the need for ongoing monitoring of local resistance trends to improve patient outcomes and guide more effective therapeutic choices in routine practice.

## Figures and Tables

**Table 1:** Genotypes of *H. pylori* specimens detected by GenoType HelicoDR for CLA.

Genotype	No. of Specimens		%
	Naive	Previously Treated	
*23S* WT (positions 2146–2147)	29	11	70.2%
*23S* MUT3 (A2147G)	7	10	29.8%
Total	36	21	100%

**Table 2:** Genotypes of *H. pylori* specimens detected by GenoType HelicoDR for FQ.

Genotype	No. of Specimens		%
	Naive	Previously Treated	
*gyr* 87WT 1-4 + *gyr*91WT	31	16	82.5%
*gyr*87MUT	3	2	8.8%
*gyr*91MUT1	1	1	3.5%
*gyr*87WT2 + *gyr*87MUT	1	0	1.7%
*gyr*87WT1 + *gyr*87MUT	0	2	3.5%
Total	36	21	100%

**Table 3:** CLA and FQ resistance rates in *H. pylori*-positive patients according to treatment status.

Group (*n*)	CLA Sensitive *n* (%)	CLA Resistant *n* (%)	FQ Sensitive *n* (%)	FQ Resistant *n* (%)	*p*-Value CLA S vs. R	*p*-Value (FQ S vs. R)	*p*-Value (Naïve vs. Treated Between Groups)
Naive to treatment	29 (80.6)	7 (19.4)	31 (86.1)	5 (13.9)	<0.0001	<0.0001	CLA R: *p* = 0.018; FQ R: *p* = 0.557
Previously treated	11 (52.4)	10 (47.6)	16 (76.2)	5 (23.8)	0.124	0.002	CLA S: *p* = 0.018; FQ S: *p* = 0.557
Total	40 (70.2)	17 (29.8)	47 (82.5)	10 (17.5)			

Data are presented as number of patients (percentage). *p*-values represent within-group comparisons of sensitivity versus resistance for each antibiotic, as well as between-group comparisons (treatment-naïve vs. previously treated). Statistical significance was defined as *p* < 0.05.

## Data Availability

The original contributions presented in this study are included in the article. Further inquiries can be directed to the corresponding author.
